# Layer-specific fast strain-encoded cardiac magnetic resonance imaging aids in the identification and discrimination of acute myocardial injury: a prospective proof-of-concept study

**DOI:** 10.1016/j.jocmr.2024.101001

**Published:** 2024-01-19

**Authors:** Lukas D. Weberling, David Albert, Andreas Ochs, Marco Ochs, Deborah Siry, Janek Salatzki, Evangelos Giannitsis, Norbert Frey, Johannes Riffel, Florian André

**Affiliations:** a Department of Cardiology, Angiology and Pneumology, Heidelberg University Hospital, Heidelberg, Germany; b DZHK (German Centre for Cardiovascular Research), Heidelberg, Germany; c Department of Cardiology, Angiology, Frankfurt University Hospital, Frankfurt am Main, Germany; d Faculty of Medicine, University of Heidelberg, Heidelberg, Germany; e Department of Cardiology and Angiology, Robert-Bosch-Hospital, Stuttgart, Germany

**Keywords:** CMR, Cardiovascular Imaging, NSTEMI, Myocarditis, fSENC, Strain

## Abstract

**Background:**

Acute myocardial injury is a common diagnosis in the emergency department and differential diagnoses are numerous. Cardiac magnetic resonance (CMR) strain sequences, such as fast strain ENCoded (fSENC), are early predictors of myocardial function loss. This study assessed the potential diagnostic and prognostic benefits of a layer-specific approach.

**Methods:**

For this prospective study, patients in the emergency department fulfilling rule-in criteria for non-ST-elevation myocardial infarction (NSTEMI) received an ultra-fast fSENC CMR. Volunteers without cardiac diseases (controls) were recruited for comparison. Measurements were performed in a single heartbeat acquisition to measure global longitudinal strain (GLS) and segmental longitudinal strain and dysfunctional segments. The GLS was measured in two layers and a difference (GLS_difference_ = GLS_epicardial_ – GLS_endocardial_) was calculated. The performance of those strain features was compared to standard care (physical examination, cardiac biomarkers, electrocardiogram). According to the final diagnosis after discharge, patients were divided into groups and followed up for 2 years.

**Results:**

A total of 114 participants, including 50 controls, were included. The 64 patients (51 male) were divided into a NSTEMI (25), myocarditis (16), and other myocardial injury group (23). GLS served as a potent predictor of myocardial injury (area under the curve (AUC) 91.8%). The GLS_difference_ provided an excellent diagnostic performance to identify a NSTEMI (AUC 83.2%), further improved by including dysfunctional segments (AUC 87.5%, p = 0.01). An optimal test was achieved by adding fSENC to standard care (AUC 95.5%, sensitivity 96.0%, specificity 86.5%, p = 0.03). No death occurred in 2 years for patients with normal GLS and ≤5 dysfunctional segments, while three patients died that showed abnormal GLS or >5 dysfunctional segments.

**Conclusions:**

Layer-specific strain is a potential new marker with high diagnostic performance in the identification and differentiation of acute myocardial injuries.

## Graphical abstract

**Figure:**
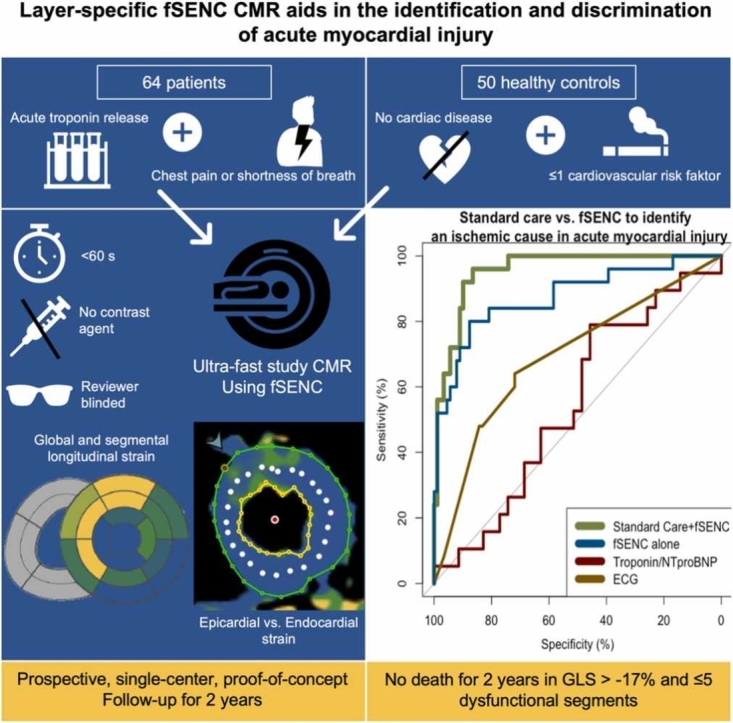

## Background

Diagnosing an acute myocardial injury is common in the emergency department (ED), ascribable to the improved identification through high-sensitivity troponin assays [1]. However, differential diagnoses of underlying causes are numerous and treatment options vary widely [2]. Invasive treatment options, such as coronary angiography, involve inherent risks and their use should be limited to necessary cases. In clinical practice, differentiating between diagnoses needing invasive procedures (such as myocardial infarction) or non-invasive treatment (such as myocarditis) is often demanding. Thus, additional non-invasive diagnostical options to guide treatment are required.

Myocardial strain has improved the assessment of myocardial function in cardiac imaging. Myocardial strain describes the deformation of the cardiac wall during the cardiac cycle and allows for the separate evaluation of longitudinal/circumferential wall shortening and radial wall thickening [3]. In addition, strain assessment is an early predictor of cardiotoxicity in cancer patients, heart failure in at-risk patients, and death in ischemic or dilatative cardiomyopathy, with incremental prognostic and diagnostic value over ejection fraction or late gadolinium enhancement [4], [5], [6], [7], [8].

Most clinically relevant strain measurement techniques track specific patterns within an image, which is called speckle tracking in echocardiography (STE) or feature tracking (FT) in cardiovascular magnetic resonance (CMR). However, these image-derived strain techniques largely depend on the quality of the assessed image and vary significantly between different software, observer, and vendor [3], [9], [10]. CMR alone offers an alternative, in which strain is measured by the physical properties of the tissue itself through an approach called CMR tagging. It has been widely accepted as the reference standard for strain quantification and shows excellent inter- and intraobserver variability [3], [11]. In general, derived techniques, such as harmonic phase imaging, Displacement Encoding with Stimulated Echos, or Strain Encoding (SENC), are available for that purpose [3].

An advancement of the SENC-technique called fast-SENC (fSENC) has enabled the assessment of myocardial strain in under 60 s, making it a promising tool for the ED [12], [13].

The various causes of acute myocardial injury affect different portions of the myocardium. For example, the inflammation in myocarditis is predominantly subepicardial. In contrast, a non-ST-elevation myocardial infarction (NSTEMI) mainly damages the subendocardial cells (due to collateral blood flow in the subepicardium) [14], [15].

In theory, the spatial resolution of fSENC (<2 mm) should be capable of differentiating those subepicardial from subendocardial pathologies [3]. In a proof-of-concept approach, this study aims to assess the diagnostic and prognostic predictive power of layer-specific strain in patients with suspected ischemic causes.

## Methods

### Study population and design

Participants were prospectively enrolled for 18 months. Our ED was screened for patients fulfilling the rule-in criteria of NSTEMI as defined by the current and previous European Society of Cardiology guidelines [16]. A high-sensitivity troponin T (hsTnT) with a fast 1-h algorithm was used as a standard of care in our ED. For our assay (reference <14 pg/mL), a rule-in was reached by the presence of symptoms suggestive of myocardial ischemia (shortness of breath, chest pain) and one of the following hsTnT findings: (1) an absolute change within the first hour of ≥5 pg/mL or (2) any value ≥52 pg/mL. Standard care involved a 15-lead electrocardiogram (including V7-V9), a complete physical examination, and laboratory diagnostics (0 h/1 h hsTnT, complete blood count, creatinine, potassium, sodium). When considered necessary by the treating physician, a transthoracic echocardiography, an invasive angiography, a full clinical CMR, or a computed tomography were performed. For all patients, an ultra-fast study CMR was conducted no later than 24 h after the event and before invasive treatment. Patient treatment (e.g., invasive coronary angiography) was always prioritized and no patient’s treatment was delayed due to participation in the study. After discharge from the ED or the inpatient ward, the final diagnosis of the treating cardiologist was noted and patients were divided into three groups: myocarditis, NSTEMI, and other myocardial injury (OMI). The diagnosis of an NSTEMI was based upon the evidence of a severe coronary stenosis on invasive coronary angiography and additionally on supporting evidence from echocardiography, whereas the diagnosis of a myocarditis was based on a full clinical CMR scan (apart from the study scan) using the updated Lake Louise criteria for diagnosis [15]. The OMI group consisted of all patients that could not be categorized into either NSTEMI or myocarditis. For comparison, an additional group of healthy volunteers without a history, symptoms, or signs of cardiac disease (e.g., left bundle branch block) and with a maximum of one cardiovascular risk factor was recruited through public announcements. In volunteers, a study CMR was conducted for research purposes only. For 2 years after the study CMR, patients and volunteers were followed up for occurrence of cardiovascular death or death from any cause.

This prospective, single-center study was approved by the ethical committee of our university (S-156/2019) and carried out in accordance with the Declaration of Helsinki. Written informed consent was given by all patients and volunteers.

### CMR acquisition protocol

CMR examinations were performed on a clinical 1.5T or 3T MRI scanner (IngeniaCx and Ingenia, Philips Healthcare, Best, The Netherlands). fSENC was performed as a single heartbeat acquisition of three short-axis views (basal, mid, apical) to assess the longitudinal shortening as described earlier [12], [13]. To test a realistic real-world rapid-CMR that may be integrated into clinical emergency routine, the study protocol only involved fSENC images and no steady-state free precession imaging (and therefore no ejection fraction), mapping or late gadolinium enhancement to save time. A full CMR scan was performed separately when clinically indicated in selected cases (especially when myocarditis was suspected).

### Image analysis

**Fig. 1:**
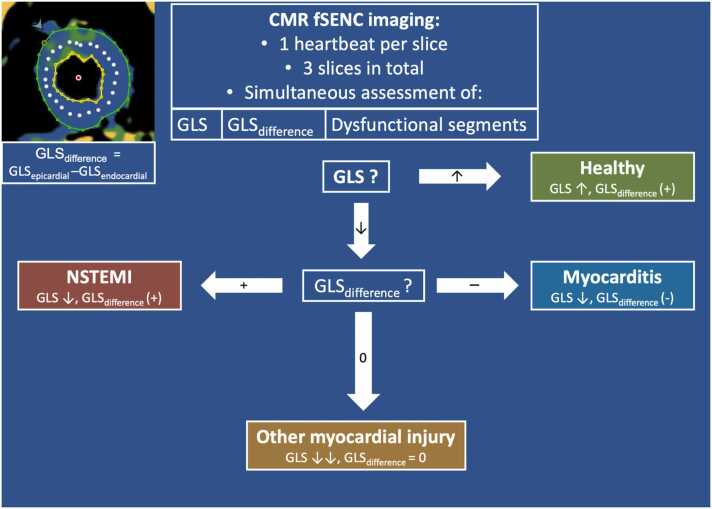
Portrayal of our suggested diagnostic algorithm to assess acute myocardial injury using fSENC. All measurements were done on three short axis slices (exemplary picture in the top left corner). GLS is measured between the epicardial (green) and endocardial (yellows) contours, GLS_Endo_ between the endocardial and mid-myocardial contour (white), and GLS_Epi_ between the epicardial and mid-myocardial contour. CMR: cardiac magnetic resonance, fSENC: fast Strain ENCoded, GLS: global longitudinal strain.

fSENC analysis was done in a separate room on a dedicated workstation by a reviewer (L.W.) with over 3 years of CMR experience, blinded to all clinical data. fSENC image quality was rated as excellent, good, adequate, or non-diagnostic. To assess inter-rater reliability, 10 randomly chosen volunteers were analyzed by another blinded reader (D.A.). Strain was measured using a dedicated analysis software (MyoStrain Version 5.2.3, Myocardial Solutions, Morrisville, North Carolina). As illustrated in Fig. 1, epicardial, endocardial, and mid-myocardial contours were drawn on all three end-systolic short-axis slices to measure the global longitudinal strain (GLS) of the whole myocardium, the GLS in the subepicardial half of the myocardium (GLS_Epi_) and the GLS in the subendocardial half of the myocardium (GLS_Endo_). The GLS_difference_ was calculated by the following formula: GLS_difference_ = GLS_Epi_ –GLS_Endo_. A positive value therefore indicated that the subendocardial strain was lower (= better) than the subepicardial strain and vice versa. The segmental GLS for each segment, according to the American Heart Association 17-segment model, was calculated by manually defining the right ventricular insertion point. The number of mildly dysfunctional (GLS > −17%) and severely dysfunctional (GLS > −10%) segments as determined by the fSENC images was calculated for each patient. These cut-offs were chosen according to the current literature [17], [18].

### Statistical analysis

Analyses were carried out using the R language and environment for statistical computing (version 4.2.1) with the user interface R Studio (version 2022.07.0/548) [19].

Normal distribution was assessed by using the Shapiro-Wilk test. Parametric variables are given as mean ± standard deviation (SD), non-parametric variables as median with interquartile range. The Welch two sample t-test was used for the comparison of two groups and the one-way analysis of variance (ANOVA) for the comparison of several groups, when parameters were distributed normally. For multiple testing, p values were adjusted using the Bonferroni correction. Not normal distributed variables were tested for differences using the nonparametric Wilcoxon rank-sum test. Correlations between parameters were assessed using the Pearson (normally distributed) or Spearman rank (not normally distributed) correlation coefficient and correlations were classified as weak (coefficient 0.1–0.29), intermediate (0.3–0.49), or strong (>0.5). To predict the binomial value healthy (volunteers) vs. not healthy (all patients), a logistic regression with three generalized linear models was fitted which included GLS (model 1), the GLS_difference_ (model 2), or a combination of GLS_difference_ and the number of mildly and severely dysfunctional segments (model 3). The same three models were fitted using a logistic regression to predict the diagnosis of an NSTEMI vs. not NSTEMI (myocarditis, OMI, healthy). All models were adjusted for age and gender. Receiver operating characteristic analyses were used to assess the test accuracy for each model. A Cox proportional-hazards model was employed to investigate the association of survival time and strain-derived parameters. For inter-rater reliability, the two-way random effects intraclass correlation coefficient (ICC 2,1) was calculated. Statistical significance was assumed at p < 0.05.

## Results

### Study population

A total of 114 participants (64 patients, 50 controls) were included in this study.

The patient cohort reflects typical cardiovascular risk factors as it is male dominated (51/64, 79.7%) and of advanced age (64.1 ± 16.8 years). For most patients (62.5%, 40/64), the ED presentation was the first cardiac event ever. Angina symptoms were most common in the NSTEMI group (88.0%, 22/25, p = 0.004). On a descriptive level, the occurrence of dyspnea, as well as the peak hsTnT and N-terminal pro-B-type natriuretic peptide (NTproBNP) levels were unequally distributed, but these did not reach significance. Nearly all patients had also undergone transthoracic echocardiography (93.8%, 60/64) and a majority an additional invasive coronary angiography (78.1%, 50/64).

The healthy control group was equally distributed between genders (25/25) with an age of 50.9 ± 17.7 years.

Table 1 summarizes the characteristics of the patient cohort. As described before, the OMI group consisted of patients whose diagnosis was neither NSTEMI nor myocarditis. The most common of those were acute decompensation of heart failure (7 patients), acute decompensation of severe aortic stenosis (3 patients), or takotsubo cardiomyopathy (3 patients). The individual final diagnosis of the OMI group and the most relevant diagnostic findings leading to it can be found in the Supplemental Table 1.

**Table 1:** Characteristics of the patient cohort.

		**All**	**Myocarditis**	**OMI**	**NSTEMI**	**Group difference**
**Number of patients**		64	16	23	25	-
**Gender (male/female)**		51/13	13/3	17/6	21/4	p=0.686
**Age**		64.1±16.8	55.1±17.8	65.7±17.6	68.4±13.7	p=0.037*
**First cardiac event ever**		40 (62.5%)	12 (75.0%)	11 (47.0%)	17 (68.0%)	p=0.180
**Sinus rhythm on ECG**		57 (89.1%)	15 (93.8%)	20 (87.0%)	22 (88.0%)	p=0.789
**ECG: ST/T-abnormalities**		40 (62.5%)	11 (62.5%)	14 (60.9%)	15 (60.0%)	p=0.842
ECG: ST-Elevation or LBBB or RBBB**		14 (21.9%)	7 (43.8%)	4 (17.4%)	3 (12.0%)	p=0.045*
**Angina symptoms**		41 (64.1%)	7 (43.8%)	12 (52.2%)	22 (88.0%)	p=0.004*
**Shortness of breath**		33 (51.6%)	6 (37.5%)	14 (60.9%)	13 (52.0%)	p=0.367
**Peak hsTnT, pg/ml**		81 (172)	239 (235)	58 (30)	84 (166)	p=0.315
**NTproBNP, ng/l (available for 54 of 64 patients)**		741 (3580)	824 (2808)	2889 (5735)	458 (3356)	p=0.434
**Cardiovascular death at 2-year follow-up**		3 (4.7%)	2 (12.5%)	1 (4.3%)	0 (0.0%)	p=0.187
**Available diagnostic work-up**						
	**15-lead ECG, physical examination, laboratory values**	64 (100.0%)	16 (100.0%)	23 (100.0%)	25 (100.0%)	
	**Echocardiography**	60 (93.8%)	16 (100.0%)	21 (91.3%)	23 (92.0%)	
	**Full CMR**	35 (54.7%)	16 (100.0%)	10 (43.5%)	9 (36.0%)	
	**Invasive coronary angiography**	50 (78.1%)	11 (68.8%)	14 (60.9%)	25 (100.0%)	

*CMR: cardiiovascular magnetic resonance; ECG: electrocardiogram; hsTnT: high-sensitivity troponin T; LBBB: left bundle branch block;* ]*NSTEMI: non-ST-elevation myocardial infarction; NTproBNP: N-terminal pro-B-type natriuretic peptide;OMI: other myocardial injury; RBBB: right bundle branch block.*

*Reaching statistical significance.

**As ST-elevation myocardial infarctions were, per study protocol, not included, these represent pre-existing LBBB/RBBB and non-significant ST-elevations.

### GLS and GLS_difference_

fSENC images were rated excellent in 48, good in 53, and adequate in 12 participants. No fSENC images were labeled non-diagnostic. Inter-rater reliability expressed using the intra-class correlation coefficient (ICC) was excellent for GLS (0.99; confidence interval (CI) 0.86–1.00), GLS_Epi_ (0.99; CI 0.96–1.00), and GLS_Endo_ (0.97, CI 0.88–0.99).

GLS was lowest (= best) in the healthy control group (−20.3 ± 1.6%), whereas it was impaired in all other groups (myocarditis −15.8 ± 3.9%; OMI −14.1 ± 4.3%; NSTEMI −14.9 ± 3.5%). A significant group difference for GLS (p < 0.001) was driven by a difference of the healthy group to all patient groups individually (p < 0.001 for healthy vs. NSTEMI [CI 6.8; 3.8], healthy vs. OMI [CI 8.1; 4.3], healthy vs. myocarditis [CI 6.6; 2.4], healthy vs. patients [CI 6.5; 4.4]). No statistically significant difference was found between patient groups for GLS (p > 0.05 for all tests).

Equally, the number of mildly and severely dysfunctional segments were lowest in the healthy control group with a statistical difference to each patient group individually (p < 0.001) and no statistical difference between patient groups (p > 0.05 for all tests).

**Fig. 2:**
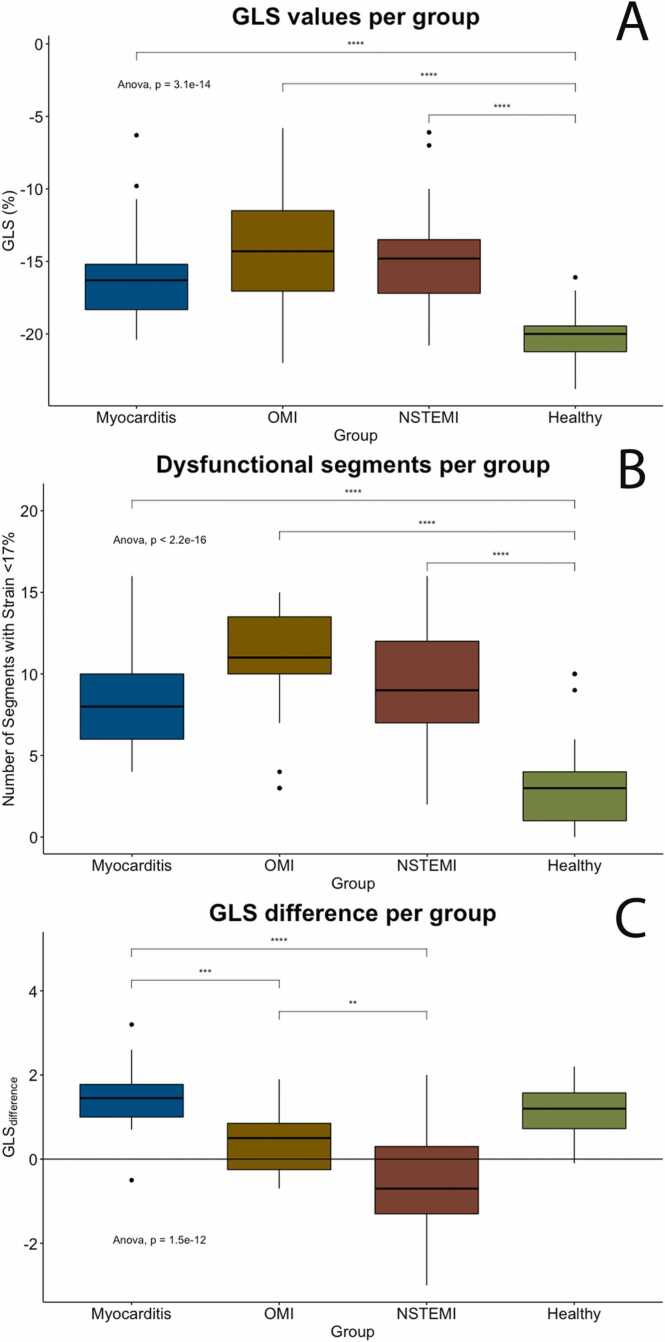
Boxplots of the GLS (A), the number of segments with mildly dysfunctional GLS (B), and the GLS_difference_ (C) per group. ANOVA test results are given in each plot. Bars above boxplots show significance levels. GLS (A) and number of mildly dysfunctional GLS (B) vary significantly between healthy controls and each patient group but lacks discriminatory power to differentiate between different pathological entities. GLS_difference_ (C) on the other hand significantly varies between the patient groups. ANOVA: analysis of variance, GLS: global longitudinal strain, NSTEMI: non-ST-elevation myocardial infarction, OMI: other myocardial injury.

**Fig. 3:**
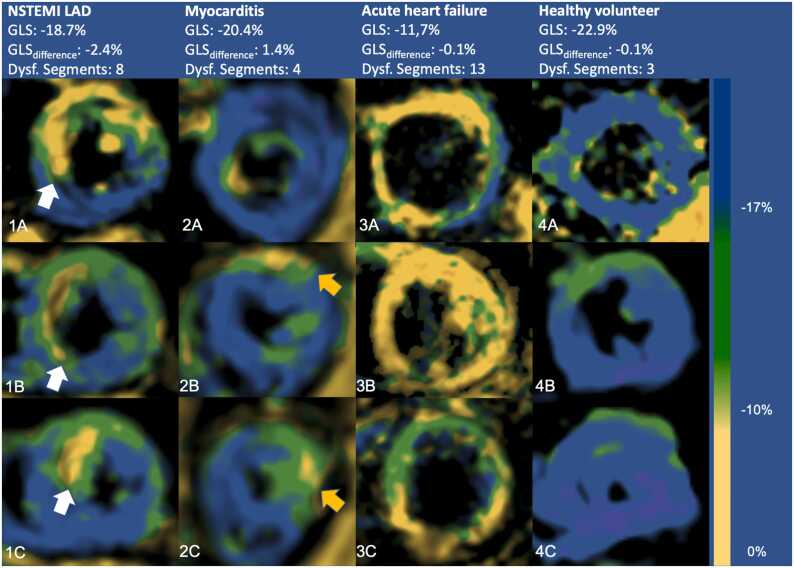
Exemplary fSENC color-coded GLS measurements in the basal (A), mid-ventricular (B), and apical (C) slice of three patients (1–3) and one healthy volunteer (4). Patient 1 had a NSTEMI with a sub-total stenosis of the LAD that later was treated by stent implantation. A subendocardial accentuation of decreased GLS is visible (white arrows). Patient 2 suffered from a myocarditis as confirmed by clinically indicated CMR and did not undergo stent implantation. Here, a subepicardial accentuation of impaired GLS in the lateral wall is seen (orange arrows). Opposed to that, patient 3 suffering from acute cardiac decompensation and a healthy volunteer (4) show neither subepicardial nor subendocardial accentuation of impaired GLS values. The overall GLS is helpful to differentiate patients (1–3) from healthy volunteers. fSENC: fast Strain ENCoded, GLS: global longitudinal strain, LAD left anterior descedingt artery, NSTEMI: non-ST-elevation myocardial infarction.

For GLS_difference_ on the other hand, values did differ significantly between patient groups. The NSTEMI group had a negative GLS_difference_ (−0.5 ± 1.3), indicating an impaired strain predominantly in the subendocardial layers of the myocardium. The GLS_difference_ of the NSTEMI group was significantly lower than the myocarditis (p < 0.001, CI −1.3; −2.7), OMI (p = 0.003, CI −0.3; −1.6), and healthy control group (p < 0.001, CI −1.1; −2.2). In contrast, the GLS_difference_ of the myocarditis group was positive (1.5 ± 0.9), a correlate of an impaired strain predominantly in the subepicardial layer. The GLS_difference_ was also positive for the OMI group (0.4 ± 0.7) and the healthy group (1.1 ± 0.6). Here, a statistical difference was only found between the OMI and myocarditis group (CI −1.6; −0.5), but not between the myocarditis group and healthy controls (p = 0.12). The fSENC-derived strain parameters are displayed in Tables 2 and 3 as well as in Fig. 2. Fig. 3 shows representative fSENC maps of each group.

**Table 2:** Overview of the used fSENC-derived parameters for each of the compared groups as well as the measurements (including feature tracking strain) derived from the full clinical CMR scans in available patients.

**fSENC-derived parameters per group**				
	**Myocarditis**	**OMI**	**NSTEMI**	**Healthy**
**Scanner (1.5 T/3.0 T)**	10/6	11/12	15/10	35/15
**fSENC GLS (%)**	-15.8±3.9	-14.1±4.3	-14.9±3.5	-20.3±1.6
**fSENC GLS difference (%)**	1.5±0.9	0.4±0.7	-0.5±1.3	1.1±0.6
**fSENC number of mildly dysfunctional segments (>-17%)**	8.6±3.5	10.7±3.7	9.8±3.6	3.1±2.4
**fSENC number of severely dysfunctional segments (>-10%)**	2.8±4.0	4.5±3.8	3.6±3.5	0.2±0.5

**Subgroup: Measurements of full clinical CMR**				
	**Myocarditis**	**OMI**	**NSTEMI**	**Reference values**
**Number of full CMRs available**	16	10	9	
**Feature Tracking GLS (%)**	-12.7±3.7	-12.2±5.2	-12.6±4.3	− 17.0 ± 1.9
**Feature Tracking GLS difference (%)**	0.7±0.7	0.5±0.6	0.4±0.5	not available
**T1 mapping times at 1.5T (ms)**	1100±94	1055±45	1056±59	994±18
**T1 mapping times at 3T (ms)**	1297±32	1291±52	1289±71	1223±23
**T2 times (ms)**	57±12	54±6	55±7	49±3
**Late Gadolinium Enhancement: number of affected segments**	2.7±2.3	3.8±4.0	2.1±1.5	0

*CMR cardiiovascular magnetic resonance, fSENC fast* S*train ENCoded, GLS global longitudinal strain, NSTEMI non-ST-elevation myocardial infarction, OMI other myocardial injury, SD standard deviation.*

A comparison of the full clinical CMR to internal (mapping) or external reference values is given [42]. Values are reported as mean ± SD.

**Table 3:** Results of the fSENC-derived strain measurements per group when only patients with no prior cardiac events were included and when only patients with prior cardiac events were included.

**Subgroup: No prior cardiac events**			
	**Myocarditis**	**OMI**	**NSTEMI**
**fSENC GLS (%)**	-15.3±4.3	-17.0±3.1	-15.3±3.9
**fSENC GLS difference (%)**	1.4±0.8	0.7±0.8	-0.8±1.2
**fSENC number of mildly dysfunctional segments (>-17%)**	9.2±3.7	8.5±3.7	9.3±4.0
**fSENC number of severely dysfunctional segments (>-10%)**	3.3±4.5	2.2±2.2	3.4±4.0

**Subgroup: Prior cardiac events**			
	**Myocarditis**	**OMI**	**NSTEMI**
**fSENC GLS (%)**	-17.2±2.3	-11.5±3.5	-14.1±2.1
**fSENC GLS difference (%)**	1.8±1.0	0.2±0.6	-0.1±1.4
**fSENC number of mildly dysfunctional segments (>-17%)**	7.0±2.4	12.8±2.1	11.0±2.5
**fSENC number of severely dysfunctional segments (>-10%)**	1.0±1.4	6.7±3.7	3.9±2.3

*fSENC fast* S*train ENCoded, GLS global longitudinal strain, NSTEMI non-ST-elevation myocardial infarction, OMI other myocardial injury, SD standard deviation.*

Values are reported as mean ± SD.

In a sub-group of 35 patients, a full clinical CMR was carried out independent to the study CMR. FT GLS, T1 times, T2 times, and late gadolinium enhancement showed abnormal measurements in all patient groups, but no statistical difference was found between the groups for neither of the clinical parameters (p > 0.05 for all). More details are given in Table 2. GLS derived with FT correlated strongly with the values derived from fSENC (coefficient 0.81, p < 0.001), but multi-layer strain (GLS_difference_) did not correlate significantly between the two methods FT and fSENC (p = 0.99). fSENC GLS also correlated strongly with LV ejection fraction (coefficient −0.69, p < 0.001) and T1 values at 1.5T (coefficient 0.55, p = 0.006). There was no correlation between fSENC GLS and T1 times at 3T, T2 times or late gadolinium enhancement (p > 0.05 for all).

### Identification of myocardial injury and NSTEMI using fSENC

In the distinction between patients and healthy volunteers, the GLS alone (model 1) provided excellent test performance to predict myocardial injury with an area under the curve (AUC) of 91.8%. At a cut-off value of 17.9% (Youden index), the sensitivity was 81.3% with a specificity of 94.0%. GLS_difference_ alone (model 2) performed acceptable (AUC 69.6%), but with an apparent lack of sensitivity (sensitivity 40.6%; specificity 94.0%; cut-off −0.05%). However, adding the number of mildly and severely dysfunctional segments to the GLS_difference_ (= model 3) improved the performance significantly (p < 0.001 compared to model 2) and made it equivalent to GLS alone (AUC 94.3%, sensitivity 85.9%; specificity 94.0%, p = 0.08). The models remained significant after adjusting for age and gender. Additionally, it remained significant when patients with prior cardiac events were excluded.

In differentiating NSTEMI patients from all other patients, standard care performed acceptable with an AUC of 69.2% for electrocardiogram and 54.7% for cardiac biomarkers (hsTnT, NTproBNP). The fSENC-derived model 1 (GLS alone) performed acceptable (AUC 74.8%), but models 2 and 3 incorporating GLS_difference_ performed significantly better (model 2: AUC 83.2%, sensitivity 80.0%; specificity 76.4.0%; p = 0.04/ model 3: AUC 87.5%; sensitivity 87.6%; specificity 80.0%; p = 0.01). Models 2 and 3 remained significant after adjusting for age and gender, but model 1 with only GLS did not reach significance. All models remained significant after excluding patients with prior cardiac events. Adding the fSENC-derived imaging parameters of model 3 to the standard of care resulted in a near-optimal diagnostic accuracy (AUC 95.5%, sensitivity 96.0%, specificity 86.5%).

**Fig. 4:**
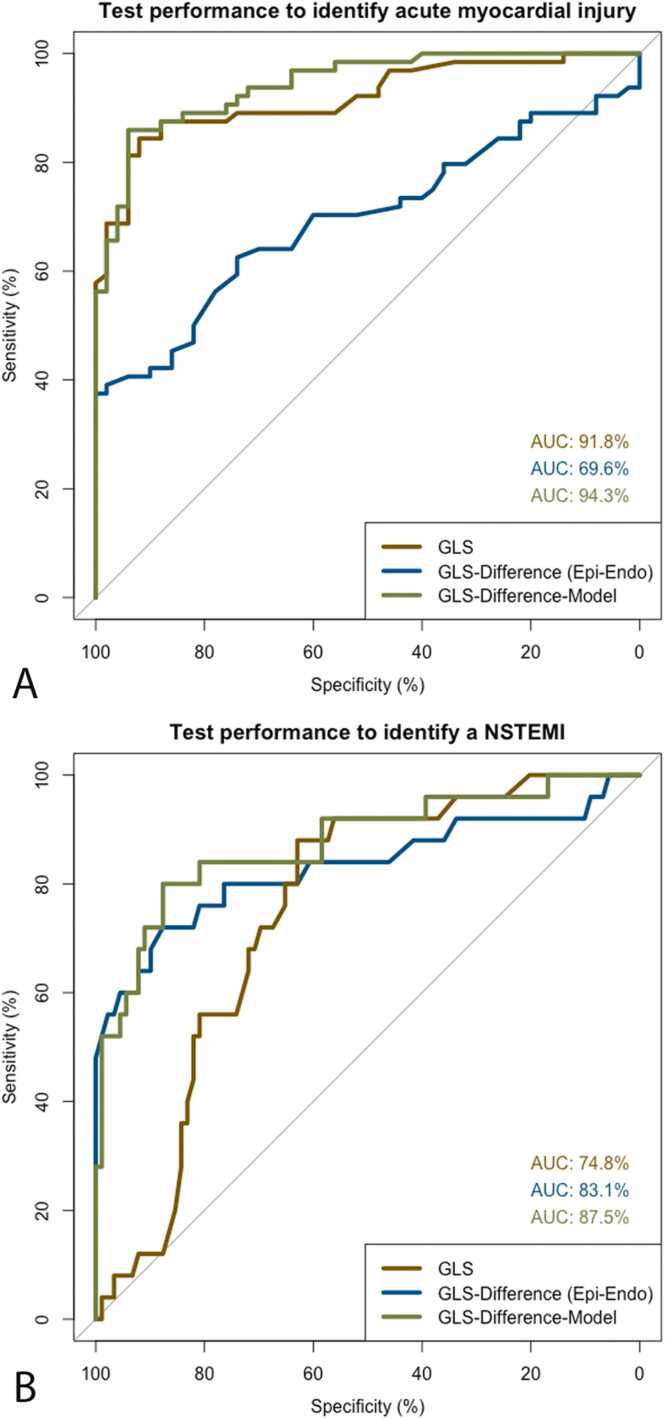
Receiver operating characteristic curves and AUC values show the performance of testing strategies in (A) the identification of myocardial injury and (B) the identification of patients with NSTEMI. The first two testing strategies included only GLS or GLS_difference_. The third strategy included a linear model incorporating GLS_difference_ and number of mildly and severely impaired segments. As shown in (B), GLS_difference_-derived testing strategies improve the identification of NSTEMI patients. AUC: area under the curve, GLS: global longitudinal strain, NSTEMI: non-ST-elevation myocardial infarction..

The performance of the three different regression models is shown in Fig. 4 and details are given in Table 4.

**Table 4:** Details of each models performance to identify an acute myocardial injury and an NSTEMI.

**Model performance to identify an acute myocardial injury**			
	**Sensitivity**	**Specificity**	**AUC**
**Model 1: fSENC GLS**	81.3%	94.0%	91.8%
**Model 2: fSENC GLS difference**	40.6%	94.0%	69.6%
**Model 3: Model 2 + dysfunctional segements**	85.9%	94.0%	94.3%

**Model performance to identify a NSTEMI**			
	**Sensitivity**	**Specificity**	**AUC**
**Model 1: fSENC GLS**	88.0%	62.9%	74.8%
**Model 2: fSENC GLS difference**	80.0%	76.4%	83.2%
**Model 3: Model 2 + dysfunctional segements**	87.6%	80.0%	87.5%
**Model 3 + Standard Care**	96.0%	86.5%	95.5%

*AUC area under the curve, fSENC fast Strain ENCoded, GLS global longitudinal strain, NSTEMI non-ST-elevation myocardial infarction*.

### Therapy and survival

Revascularization was necessary in 23 patients (46.0%) of all the 50 invasive coronary angiographies (involving 19 percutaneous coronary interventions with stent implantation and four bypass operations), of which 22 patients were in the NSTEMI group and one in the OMI (a patient with a critical aortic valve stenosis but also a 75% stenosis in the left circumflex artery).

No death occurred after 2 years of follow-up in the healthy volunteers and the NSTEMI group. Two cardiovascular deaths occurred in the myocarditis group (12.5%) and one in the OMI group (4.3%). fSENC-derived parameters (GLS, GLS_Epi_, GLS_Endo_, or GLS_difference_) did not reach significance levels in the survival time prediction. However, patients and participants with a normal GLS of <−17% and ≤5 mildly dysfunctional segments showed a 100% 2-year survival rate.

## Discussion

Our study shows an incremental diagnostic value of a layer-specific strain approach in assessing ED patients with acute myocardial injury. To the best of our knowledge, it is the first study using layer-specific strain to discriminate between different pathological entities in an acute setting.

Our data suggest a stepwise approach for the differential diagnosis in suspected acute myocardial injury and a graphical illustration of this approach is given in Fig. 1. In a first step, GLS may be used to differentiate healthy hearts from acute myocardial injuries. Here, our study showed excellent discriminatory power (AUC 91.8%). In a second step, the GLS_difference_ aids in the identification of the underlying pathology. Our healthy controls exhibited a positive GLS_difference_, a correlate of lower GLS values in the subendocardium. This is supported by postmortem studies of the human heart showing the subendocardial region to consist of mainly longitudinally directed fibers and other imaging studies assessing a layer-specific GLS in healthy controls [6], [20], [21], [22], [23], [24]. In myocarditis patients, the GLS_difference_ remained positive, presumably caused by a decrease of the epicardial strain through local edema [15]. The combination of decreased strain and positive GLS_difference_ was unique for myocarditis patients and facilitates myocarditis diagnosis. On the other hand, the OMI group had a combination of decreased strain and a GLS_difference_ indifferent to zero. Looking at the diagnoses combined in this group (acute decompensation, arrhythmias, takotsubo), this can be explained by myocardial damage (therefore decreased strain) throughout the myocardium, which is not limited to a specific layer (therefore GLS_difference_ indifferent to zero). In NSTEMI patients, on the other hand, the GLS_difference_ was negative marking a higher strain in the epicardium. This supports our initial hypothesis as ischemic cell death occurs primarily in the endocardium due to reduced collateral blood-flow [14]. Again, the combination of reduced GLS and negative GLS_difference_ was unique for the diagnosis of NSTEMI. This is particularly relevant since our study observed that an invasive coronary angiography was done in 78.1% of all patients, although revascularization was only necessary in 35.9% of those (n = 23). The here proposed layer-specific GLS_difference_ stands out with a high diagnostic test performance (AUC 87.5% alone and 95.5% when added to standard care) and might aid in the discrimination of NSTEMI patients from other causes of myocardial injury and therefore limit invasive angiographies.

Other studies using strain to improve diagnostic decision making mainly focused on global strain alone. For example, they show that it may detect the presence of microvascular obstruction after STEMI, serve as alternative imaging of vasodilator stress impact, or identify heart failure with preserved ejection fraction [25], [26], [27]. The only study using a layer-specific diagnostic approach showed the STE-derived subepicardial strain to be a predictor of coronary artery disease [28]. Most studies assessing layer-specific strain values focused on outcome prediction in stable patients, limiting their comparability to our study [6], [18], [21], [22], [29], [30], [31], [32]. None of the studies evaluated the diagnostic benefit of a layer-specific approach for diagnosis as they each assessed singular pathologic entities only.

This study confirms previously published studies about the great predictive value of global strain values [4], [5], [6], [7]. In our study, no death occurred in patients with GLS <−17% and ≤5 mildly dysfunctional segments.

The 2-year survival rate of 100.0% in the NSTEMI group emphasizes the importance of revascularization in correctly diagnosed myocardial infarction patients. The high rate of treatments after the study CMR and the overall low mortality of included patients (4.7%) are in our opinion the main contributors why no fSENC-derived parameters reached levels of significance in the prediction of survival time. Two previous studies on ischemia patients assessed strain after treatment and improved prognostic information through layer-specific strain in myocardial infarction patients at a median of 2 days after percutaneous coronary intervention [33], [34]. The same was noted by Liu et al. in an STE study on 160 NSTEMI patients before intervention. Using a broader definition and consecutively more frequent occurrence of cardiac events, an impact of layer-specific strain on prognosis was observed [35].

It has to be addressed that the comparability between STE values from different vendors is limited and strain measurements are simply not interchangeable [3]. As this is true for GLS, it is even more relevant in layer-specific strain, as shown by Ünlü et al., who found significant bias among vendors for layer-specific STE [9]. Comparisons of layer-specific FT or STE with the gold standard of tissue tagging are rare. In a single study performed on 30 patients, the authors concluded that other techniques than tissue tagging are not yet applicable for clinical or research purposes [36]. We conclude that CMR strain sequences are best suited for the assessment of layer-specific strain [37].

## Limitations

This leads to the limitations of our study. First, a comparison to image-derived strain methods was beyond the scope of the current study. Regarding the extensive literature, the high accuracy of the used variation of tissue tagging is unquestioned, but it might have been of interest if other CMR techniques are sensitive to a layer-specific approach as well [17], [38], [39]. Secondly, the sample size of 16–25 patients per group was limited owing to the proof-of-concept design of this study. Despite the small sample size, an extensive statical difference was found between the groups. Identifying this novel diagnostic and prognostic imaging marker justifies initiating a large prospective trial to verify the results. Thirdly, due to the study’s design, only a certain risk group of patients was included. Since patients with hemodynamic instability or severe persisting symptoms were excluded and treatment was—by design—not delayed by CMR acquisition, patients with timely invasive coronary angiography are underrepresented in our study. However, we argue that this is a strength of our study since fSENC still provided excellent diagnostic test performance although these diagnostically “unchallenging” cases were left out. Furthermore, patients initially classified in the “observe”-zone according to the initial hsTnT were also underrepresented in our study. Those were analyzed in a study by Riffel et al. and Siry et al., although a layer-specific approach was not used [40], [41]. Moreover, the spatial resolution of the here used fSENC sequence (2 mm) might not be sufficient to detect small subendocardial deficits or thinned myocardium. Finally, as a portion of our patients (37.5%) had previous cardiac events, their prior myocardial injuries might in part contribute to the detection by our strain models. Our study consisted of a “real-world” patient collective, and we addressed this limitation with a subanalysis of patients without previous events in which the models remained significant. Nevertheless, the role of previous events needs to be addressed in future validation trials. Finally, although a comparison with established CMR-derived parameters showed a strong correlation of fSENC GLS to FT-derived GLS, LV ejection fraction, and T1 times, multi-layer strain did not correlate between the two methods. The underlying techniques of FT and fSENC differ fundamentally and it remains questionable if the FT method can accurately measure strain on a segmental or multi-layer level, an observation that was also made by a previous study [36]. However, since only a subgroup of 35 patients received a full clinical CMR and since it was mostly not performed on the same day and in part after successful therapy, this cannot be fully answered and has to be addressed in future studies.

## Conclusions

In conclusion, the current study re-affirms the prognostic value of CMR-derived strain measurements and introduces layer-specific strain measurements as a potential new marker in assessing acute myocardial injury patients. Furthermore, it provides evidence of the high diagnostic performance in identifying ischemic causes in acute myocardial injury. Therefore, it could limit unnecessary invasive procedures in the future.

## Funding

For the analysis of the fSENC images, the authors were provided with free research licenses by Myocardial Solutions. L.D.W. was supported by the Rotation Grand (D.10021788) of the 10.13039/100010447DZHK (German Centre for Cardiovascular Research). The DZHK did not have any influence on design, analysis, or interpretation of the study. For the publication fee, we acknowledge financial support by 10.13039/501100001659Deutsche Forschungsgemeinschaft within the funding program “Open Access Publikationskosten” as well as by Heidelberg University.

## Author contributions

**Deborah Siry:** Investigation, Writing – review and editing. **Janek Salatzki:** Investigation, Writing – review and editing. **Evangelos Giannitsis:** Resources, Writing – review and editing. **Norbert Frey:** Resources, Supervision, Writing – review and editing. **Johannes Riffel:** Conceptualization, Project administration, Writing – review and editing. **Florian André :** Conceptualization, Investigation, Writing – original draft, Writing – review and editing. **Lukas Damian Weberling:** Conceptualization, Data curation, Formal analysis, Funding acquisition, Investigation, Methodology, Project administration, Resources, Writing – original draft, Writing – review and editing. **David Albert:** Data curation, Investigation, Writing – review & editing. **Andreas Ochs:** Investigation, Writing – review and editing. **Marco Ochs:** Conceptualization, Investigation, Writing – review & editing. All authors read and approved the final manuscript.

## Ethics approval and consent

This study was approved by the ethical committee of our university (S-156/2019) and carried out in accordance with the Declaration of Helsinki. Written informed consent was given by all patients and volunteers.

## Declaration of competing interest

The authors declare the following financial interests/personal relationships which may be considered as potential competing interests: Lukas Weberling reports equipment, drugs, or supplies was provided by Myocardial Solutions Inc. Lukas Weberling reports article publishing charges was provided by German Research Foundation. Lukas Weberling reports financial support was provided by German Center for Cardiovascular Disease. The other authors declare that they have no known competing financial interests or personal relationships that could have appeared to influence the work reported in this paper.
