# Supplementary material for: Layer-specific fast strain-encoded cardiac magnetic resonance imaging aids in the identification and discrimination of acute myocardial injury: a prospective proof-of-concept study
Source: J Cardiovasc Magn Reson. 2024 Jan 19;26(1):101001. doi: 10.1016/j.jocmr.2024.101001 (PMC11211227; doi:10.1016/j.jocmr.2024.101001)
Supplement: Supplementary file 1 — Supplementary material [file mmc1.docx]

**Supplemental table S1**: Overview of the exact final diagnosis of patients categorized into the group “other myocardial injury” and most relevant diagnostic findings for each patient leading to that diagnosis.

| **Sex** | **Age** | **Final diagnosis** | **Most relevant diagnostic findings** |
| --- | --- | --- | --- |
| male | 67 | Acute decompensation of heart failure | NTproBNP 29488ng/l, known ichemic heart disease, exclusion of new severe coronary stenoses on invasive angiography |
| female | 87 | Acute decompensation of severe aortic stenosis | Typical echocardiography finding, exclusion of relevant coronary stenoses with cCTA |
| male | 84 | Acute decompensation of severe aortic stenosis | Typical echocardiography finding, exclusion of relevant coronary stenoses with invasive angiography |
| male | 53 | Unknown | Initial (0 hour) and 1 hour laboratory evidence of moderate troponin release, but normal levels at 3 hour troponin control |
| male | 48 | Unknown | Initial (0 hour) and 1 hour laboratory evidence of moderate troponin release, but normal levels at 3 hour troponin control |
| male | 76 | Acute decompensation of heart failure | NTproBNP 3600ng/l, known ischemic heart disease, worsened ejection fraction on echocardiography, exclusion of new severe coronary stenoses on invasive angiography |
| male | 54 | Hypertensive derailment | Blood pressure >200mHg systolic, moderate maximum troponin release (hsTnT max. 67ng/l), normal troponin levels after treatment of blood pressure |
| female | 76 | HFpEF | Typical echocardiography finding, exclusion of relevant coronary stenoses with invasive angiography |
| male | 57 | Hypertrophic cardiomyopathy | Typical CMR and echocardiography finding, exclusion of relevant coronary stenoses with invasive angiography |
| male | 72 | Acute decompensation of heart failure | NTproBNP 2889ng/l with only moderate troponin release (hsTnT max. 92ng/l), known cardiac insufficiency |
| male | 73 | Acute decompensation of heart failure | NTproBNP 3874ng/l with only moderate troponin release (hsTnT max. 52ng/l), exclusion of relevant coronary artery stenoses with invasive angiography |
| female | 86 | Acute decompensation of heart failure | NTproBNP 25673ng/l with only moderate troponin release (hsTnT max. 72ng/l), exclusion of relevant coronary artery stenoses with invasive angiography |
| male | 70 | Cardiac toxicity of chemotherapy | New potentially cardiotoxic chemotherapy, exclusion of relevant coronary stenoses with invasive angiography |
| male | 76 | Acute decompensation of severe aortic stenosis | Typical echocardiography finding, exclusion of relevant coronary stenoses with invasive angiography |
| male | 77 | Acute decompensation of heart failure | NTproBNP 2023ng/l with only moderate troponin release (hsTnT max. 27ng/l), known cardiac insufficiency |
| male | 29 | Acute renal failure | Normal echocardiography, moderate troponin release (44ng/l) |
| female | 89 | Acute decompensation of heart failure | NTproBNP 18909ng/l with only moderate troponin release (hsTnT max. 50ng/l), known cardiac insufficiency |
| male | 65 | Tachyarrhythmia | Tachyarrhythmic event, normal echocardiography and invasive coronary angiography |
| male | 26 | Tachyarrhythmia | Tachyarrhythmic event, normal echocardiography and invasive coronary angiography |
| male | 39 | Cardiac toxicity of chemotherapy | New potentially cardiotoxic chemotherapy, exclusion of relevant coronary stenoses with invasive angiography |
| female | 78 | Takotsubo cardiomyopathy | Typical echocardiography and CMR features, exclusion of relevant coronary stenoses with invasive coronary angiography |
| female | 66 | Takotsubo cardiomyopathy | Typical echocardiography and CMR features, exclusion of relevant coronary stenoses with invasive coronary angiography |
| male | 64 | Takotsubo cardiomyopathy | Typical echocardiography and CMR features, exclusion of relevant coronary stenoses with invasive coronary angiography |
